# Supplementary figures and images for: Tristetraprolin attenuates schistosomiasis-induced liver fibrosis through m⁶A-mediated regulation of TGF-β1 mRNA stability
Source: PLoS Pathog. 2026 May 13;22(5):e1014007. doi: 10.1371/journal.ppat.1014007 (PMC13189414; doi:10.1371/journal.ppat.1014007)

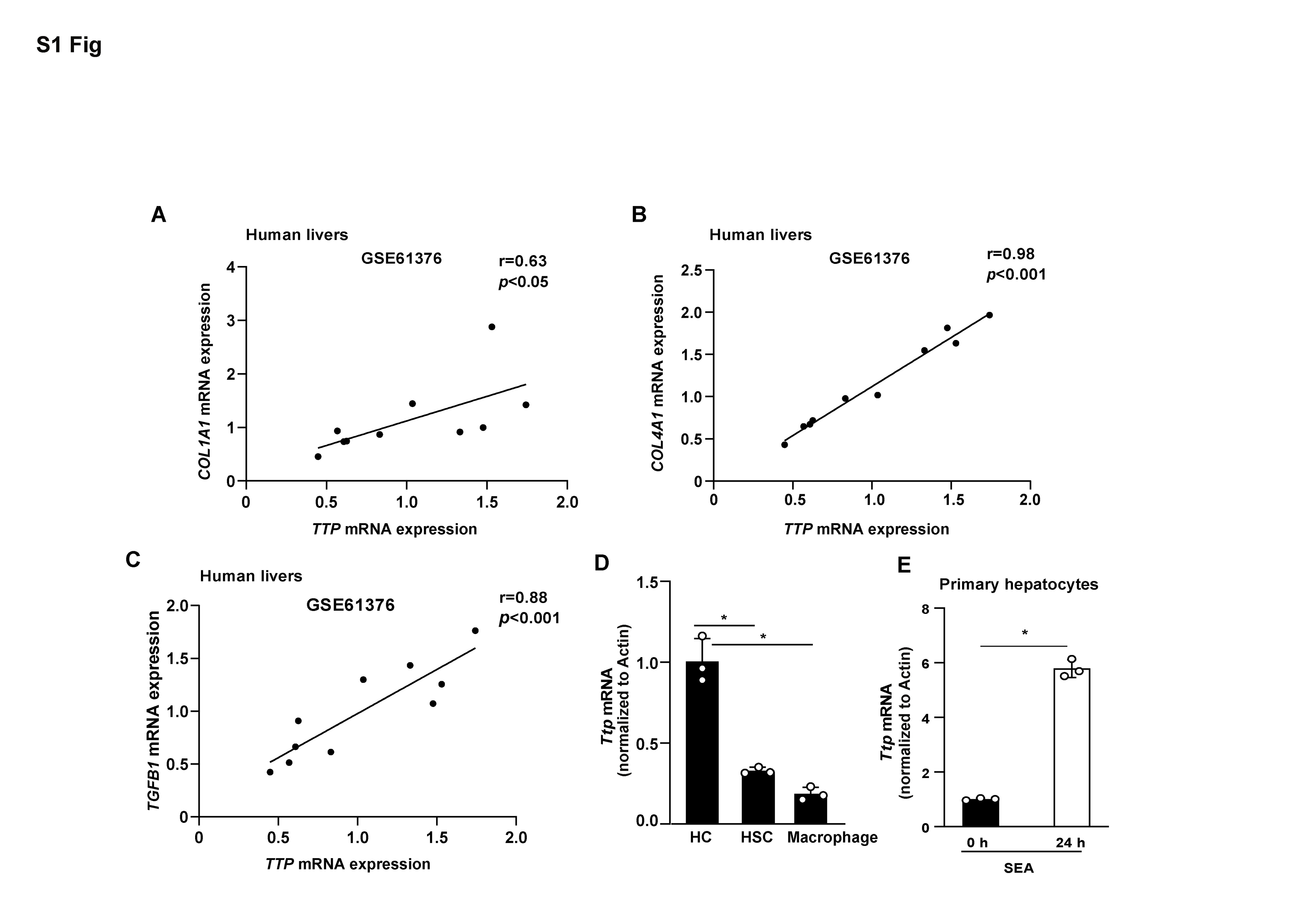

Supplement: S1 Fig — (A-C) Spearman correlation analysis was applied to assess the correlations between TTP expression and that of COL1A1 (A), COL4A1 (B), and TGFB1 (C) in human liver samples with S. japonicum-induced liver fibrosis, using data from the GSE61376 dataset. (D) The mRNA expression of TTP in hepatocytes (HC), hepatic stellate cells (HSC), and macrophages isolated from mouse liver samples. (E) Mouse primary hepatocytes were exposed to SEA (60 μg/ml) for 24 h followed by real-time PCR analysis for Ttp mRNA. Data represent mean ± SD from three independent experiments. Statistical analyses were performed using one-way ANOVA or 2-tailed Student’s t-test. *P < 0.05 versus ctrl. (TIF) [file ppat.1014007.s001.tif]

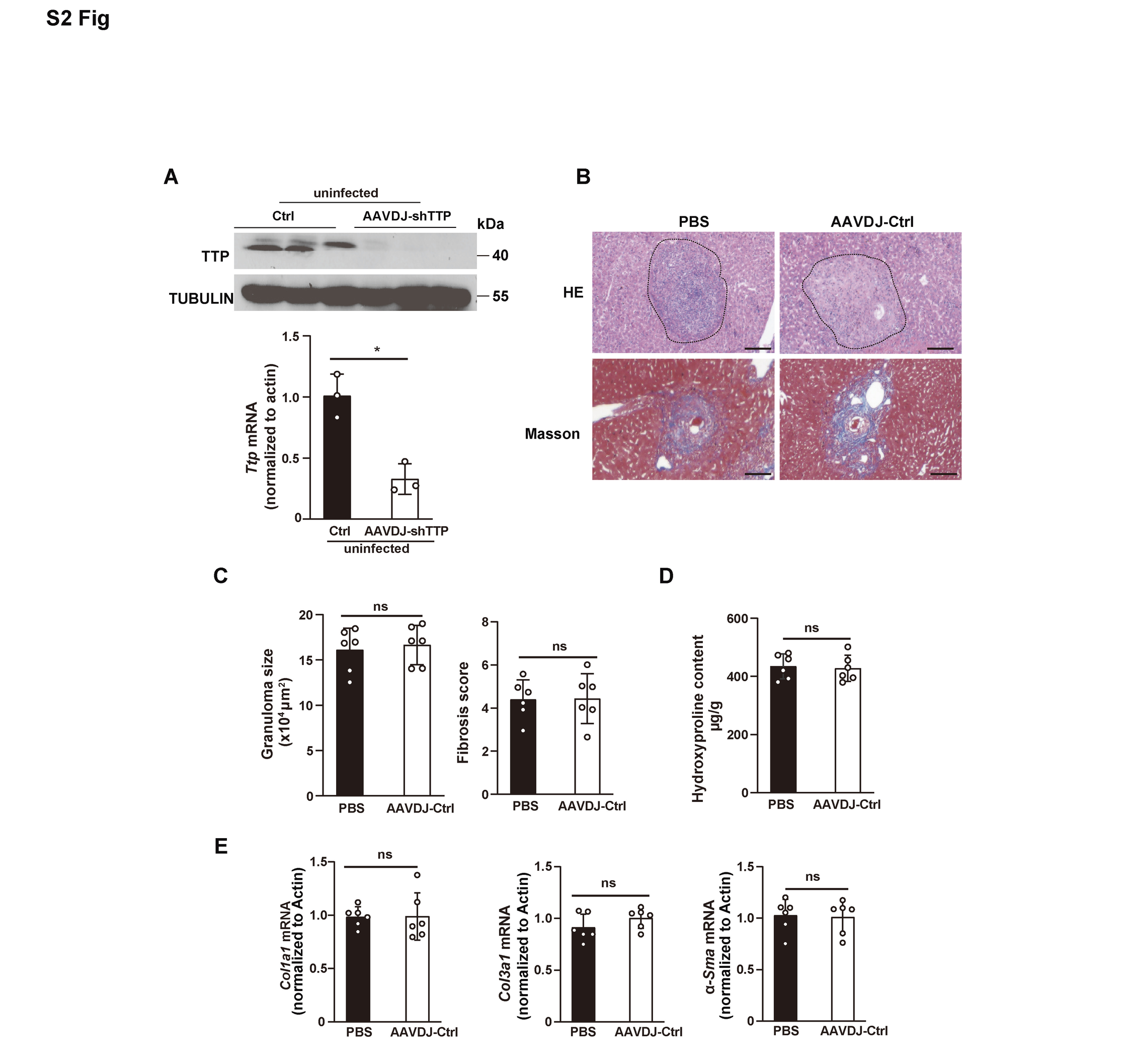

Supplement: S2 Fig — (A) Western blot (left) and real-time PCR (right) assays were conducted to assess TTP expression in liver tissues of non-infected mice following 2 weeks of AAV vector-mediated (AAVDJ-shTTP) TTP knockdown. (B) H&E staining and Masson’s trichrome staining of liver sections from the indicated groups (PBS/infected and AAVDJ-shCtrl/infected) (Scale bar: 200 μm). (C) Granuloma size was measured from H&E-stained liver sections, and fibrosis score was determined from Masson’s trichrome-stained liver sections. (D) The content of hydroxyproline in the liver was detected. (E) Real-time PCR analysis of Acta2, Col1α1, and Col3α1 expression level in liver tissues. Data represent mean ± SD from three independent experiments. Statistical analyses were performed using 2-tailed Student’s t-test. *P < 0.05 versus ctrl. ns, not significant. (TIF) [file ppat.1014007.s002.tif]

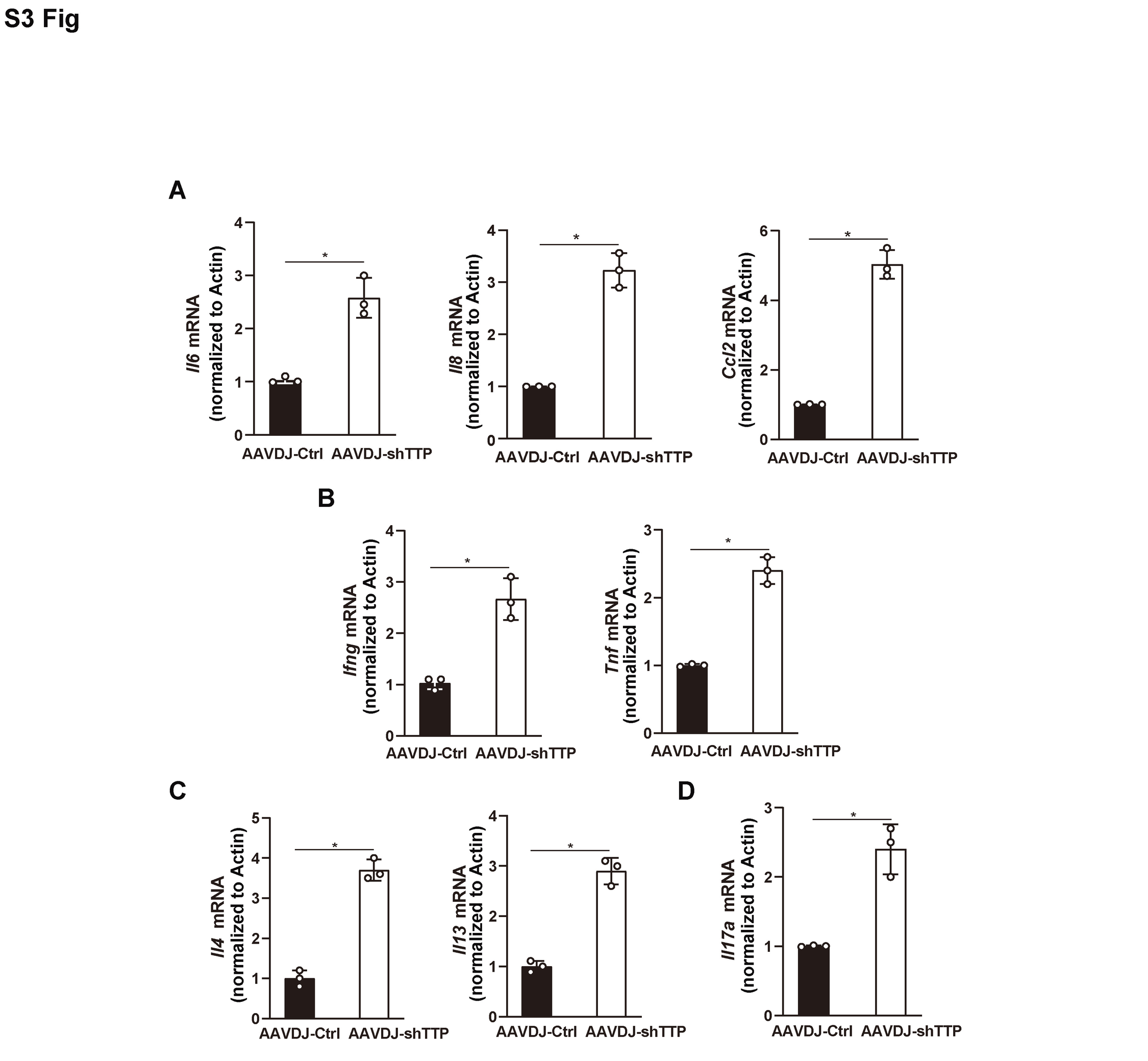

Supplement: S3 Fig — (A) Real-time PCR analysis of Il6, Il8, and Ccl2 expression level in liver tissues. (B) Real-time PCR analysis of Ifng and Tnf expression level in liver tissues. (C) Real-time PCR analysis of Il4 and Il13 expression level in liver tissues. (D) Real-time PCR analysis of Il17a expression level in liver tissues. Data represent mean ± SD from three independent experiments. Statistical analyses were performed using 2-tailed Student’s t-test. *P < 0.05 versus ctrl. (TIF) [file ppat.1014007.s003.tif]

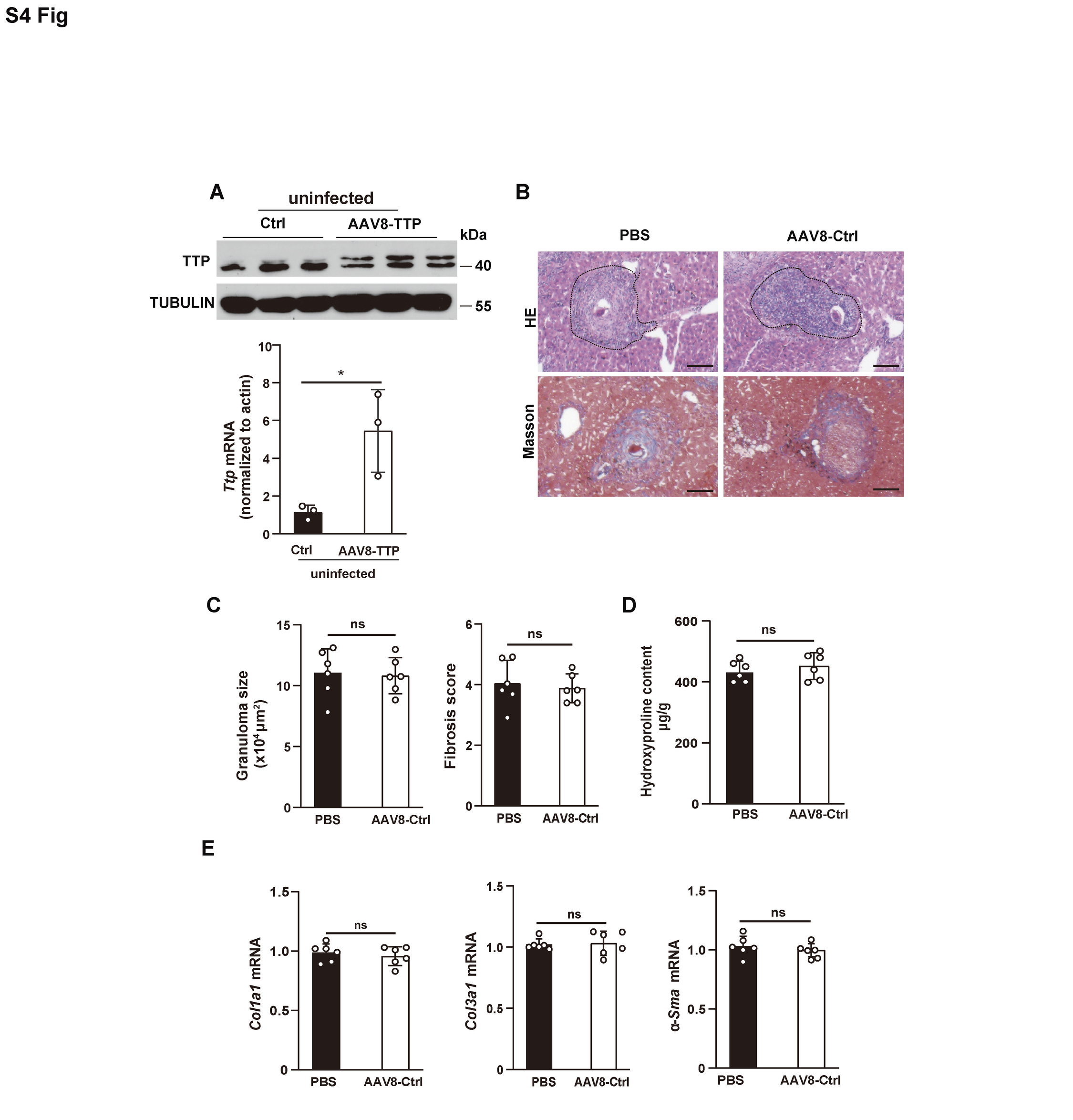

Supplement: S4 Fig — (A) Western blot (left) and real-time PCR (right) assays were conducted to assess TTP expression in liver tissues of non-infected mice following 2 weeks of AAV8 vector-mediated (AAV8-TTP) TTP overexpression. (B) H&E staining and Masson’s trichrome staining of liver sections from the indicated groups (PBS/infected and AAV8-Ctrl/infected) (Scale bar: 200 μm). (C) Granuloma size was measured from H&E-stained liver sections, and fibrosis score was determined from Masson’s trichrome-stained liver sections. (D) The content of hydroxyproline in the liver was detected. (E) Real-time PCR analysis of Acta2, Col1α1, and Col3α1 expression level in liver tissues. Data represent mean ± SD from three independent experiments. Statistical analyses were performed using 2-tailed Student’s t-test. *P < 0.05 versus ctrl. ns, not significant. (TIF) [file ppat.1014007.s004.tif]

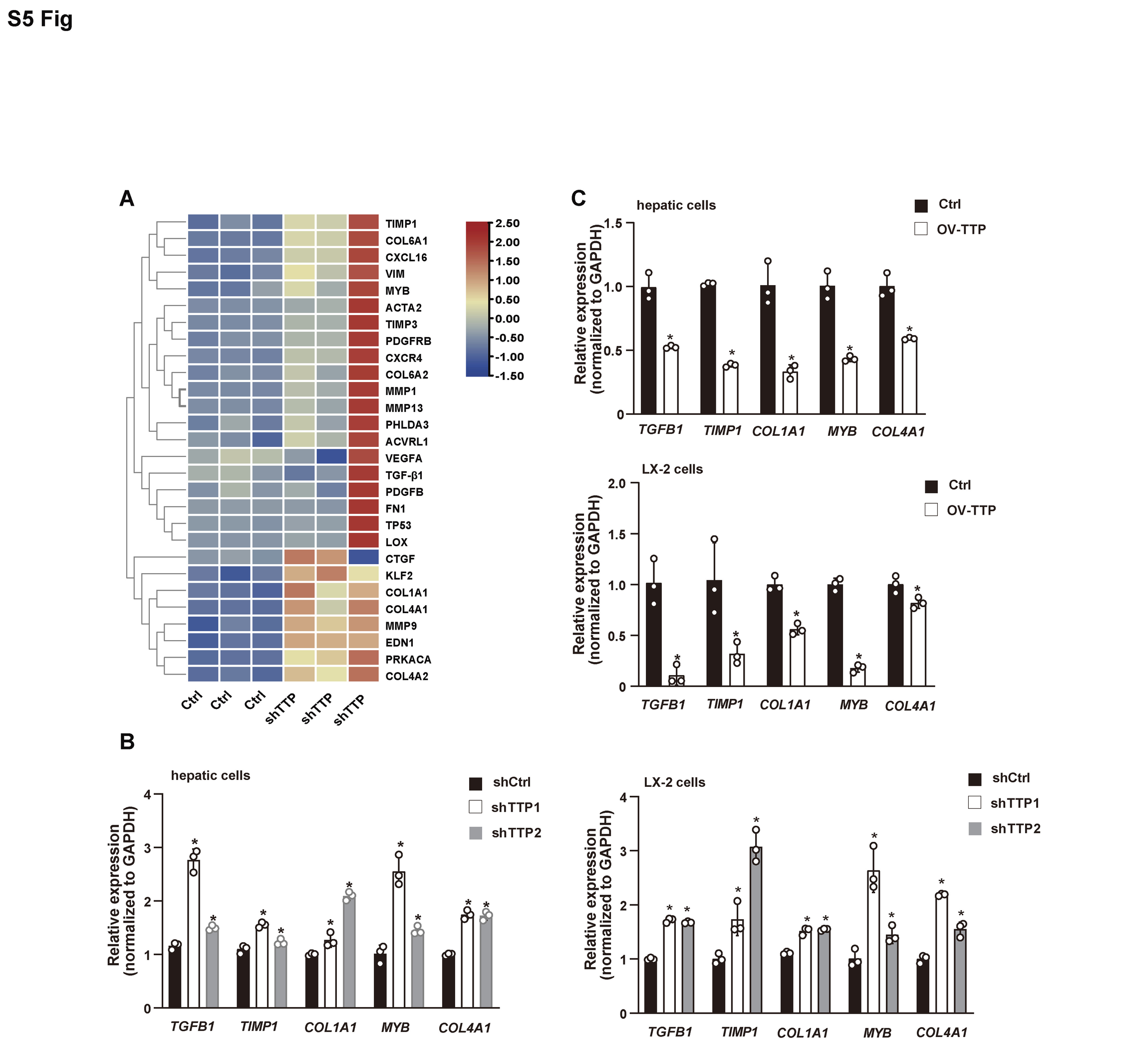

Supplement: S5 Fig — (A) The heat map illustrates the gene expression profiles of fibrosis-related cytokines in TTP-knockdown cells based on our previous RNA-seq data (GSE157581). (B) The mRNA levels of TGFB1, TIMP1, COL1A, COL4A1, and MYB were analyzed in TTP-knockdown hepatic cells or LX-2 cells via real-time PCR. (C) The mRNA levels of TGFB1, TIMP1, COL1A, COL4A1, and MYB was analyzed in TTP-overexpression hepatic cells or LX-2 cells via real-time PCR. Data represent mean ± SD from three independent experiments. Statistical analyses were performed using 2-tailed Student’s t-test and one-way ANOVA. *P < 0.05 versus ctrl. (TIF) [file ppat.1014007.s005.tif]

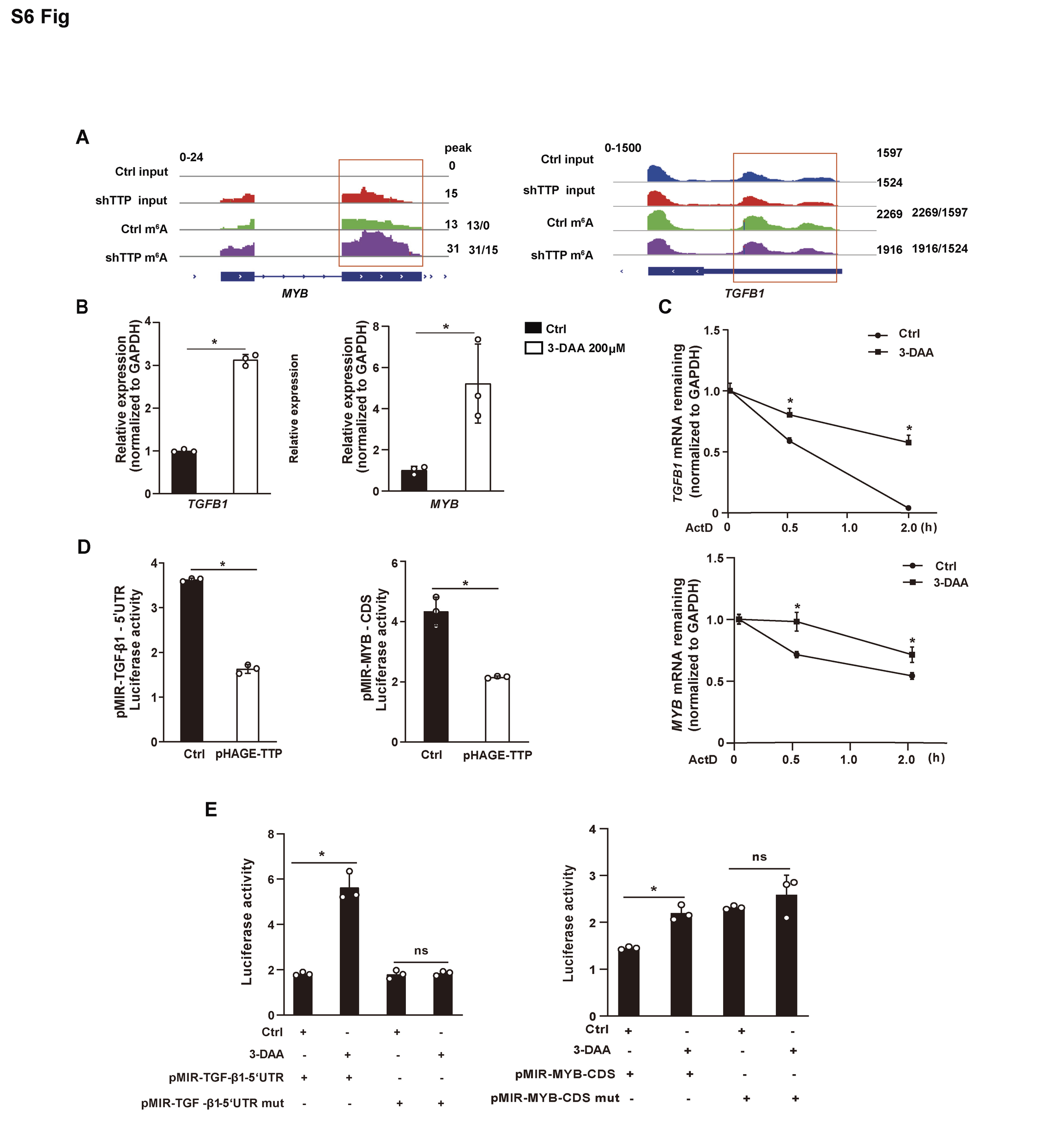

Supplement: S6 Fig — (A) An integrative genomics viewer (IGV) tracks display higher enrichment of m6A peaks in TGFB1 and MYB transcripts. (B) The mRNA levels of TGFB1 and MYB in HepG2 cells treated with 3-DAA were measured by real-time PCR. (C) The stability of TGFB1 and MYB mRNAs in hepatic cells treated with 3-DAA. (D) Analysis of luciferase levels of pMIR-TGF-β1–5’UTR, pMIR-MYB-CDS, pMIR-TGF-β1–5’UTR mut, or pMIR-MYB-CDS mut in hepatic cells treated with 3-DAA. Data represent mean ± SD from three independent experiments. Statistical analyses were performed using 2-tailed Student’s t-test and two-way ANOVA. *P < 0.05 versus ctrl. (TIF) [file ppat.1014007.s006.tif]

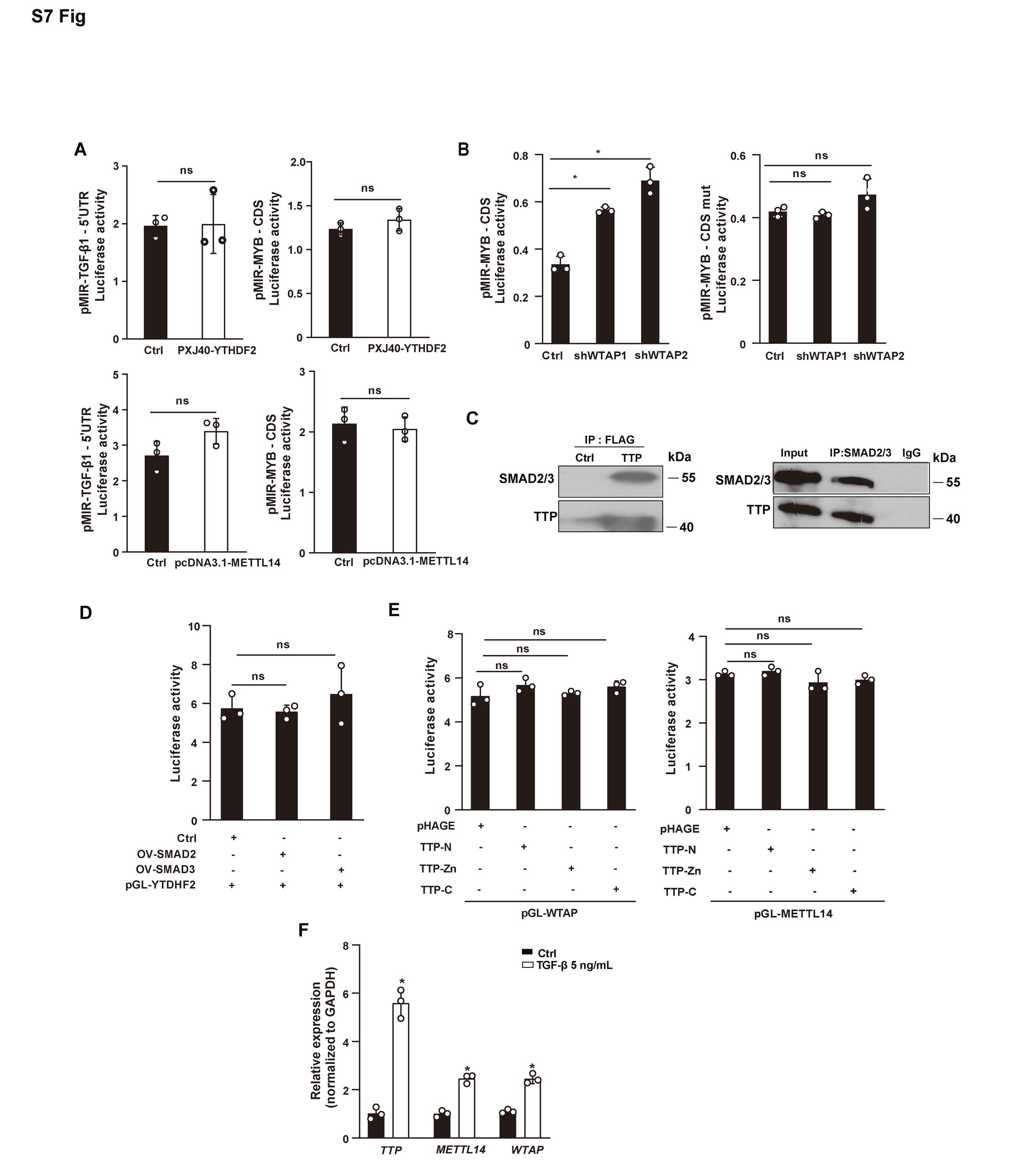

Supplement: S7 Fig — (A) Hepatic cells overexpressing METTL14 or YTHDF2 were transfected with pMIR-TGF-β1–5’UTR or pMIR-MYB-CDS, followed by luciferase analysis. (B) Stable WTAP-knockdown LX-2 cells were transfected with pMlR-MYB-CDS or pMIR-MYB-CDS mut for 24 h, followed by luciferase analysis. (C)TTP and SMAD2/3 were immunoprecipitated in HEK293T cells. (D) HEK293T cells overexpressing SMAD2/3 were transfected with pGL-YTHDF2, followed by luciferase analysis. (E) HEK293T cells were co-transfected with the promoters of WTAP or METTL14, along with TTP-N, TTP-Zn, or TTP-C, followed by luciferase analysis. (F) The expression of TTP, WTAP, and METTL14 in hepatic cells with TGF-β stimulation. Data represent mean ± SD from three independent experiments. Statistical analyses were performed using 2-tailed Student’s t-test and one-way ANOVA. *P < 0.05 versus ctrl. ns, not significant. (TIF) [file ppat.1014007.s007.tif]

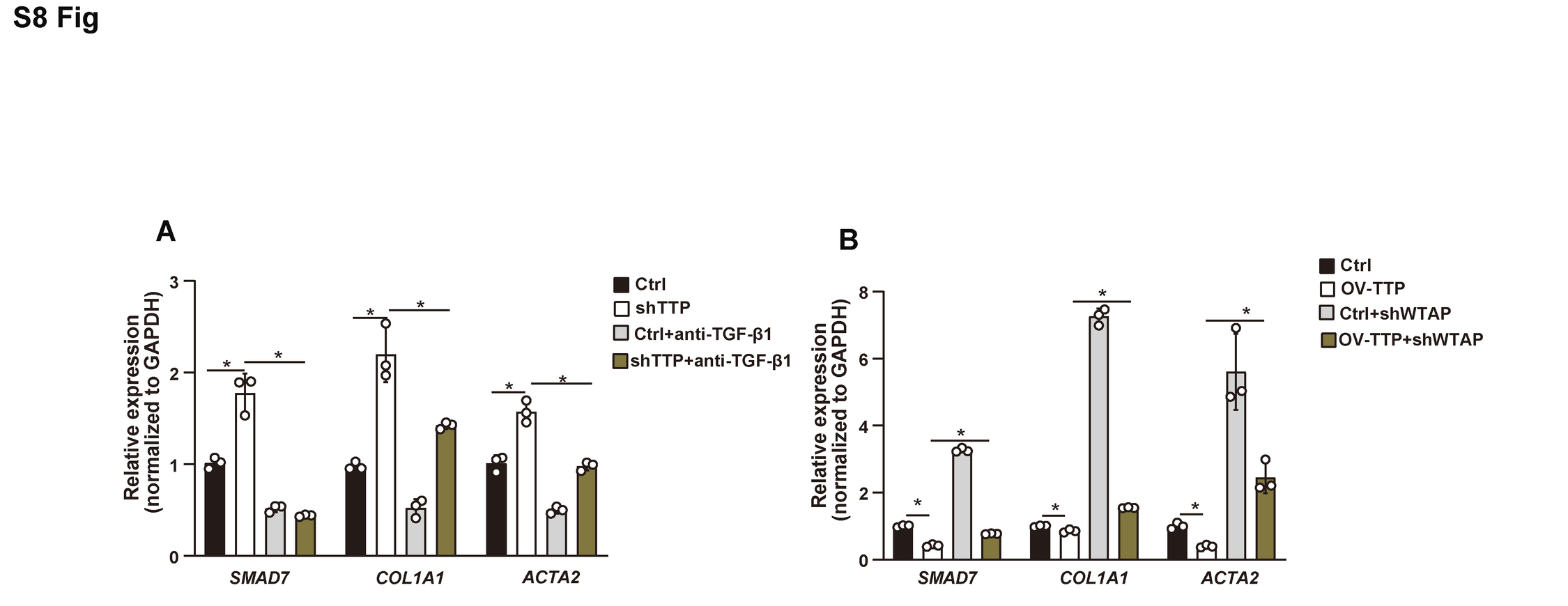

Supplement: S8 Fig — (A) The expression of SMAD7, COL1A1, and ACTA2 in LX-2 cells was analyzed by real-time PCR after 36 h of coculture with hepatic cells stably expressing shTTP, in the presence or absence of a neutralizing antibody to TGF-β1. (B) The expression of SMAD7, COL1A1, and ACTA2 was analyzed in LX-2 cells after coculture for 36 h with stably WTAP-knockdown hepatic cells transfected with TTP-overexpressing vectors. Data represent mean ± SD from three independent experiments. Statistical analyses were performed using 2-tailed Student’s t-test. *P < 0.05 versus ctrl. (TIF) [file ppat.1014007.s008.tif]

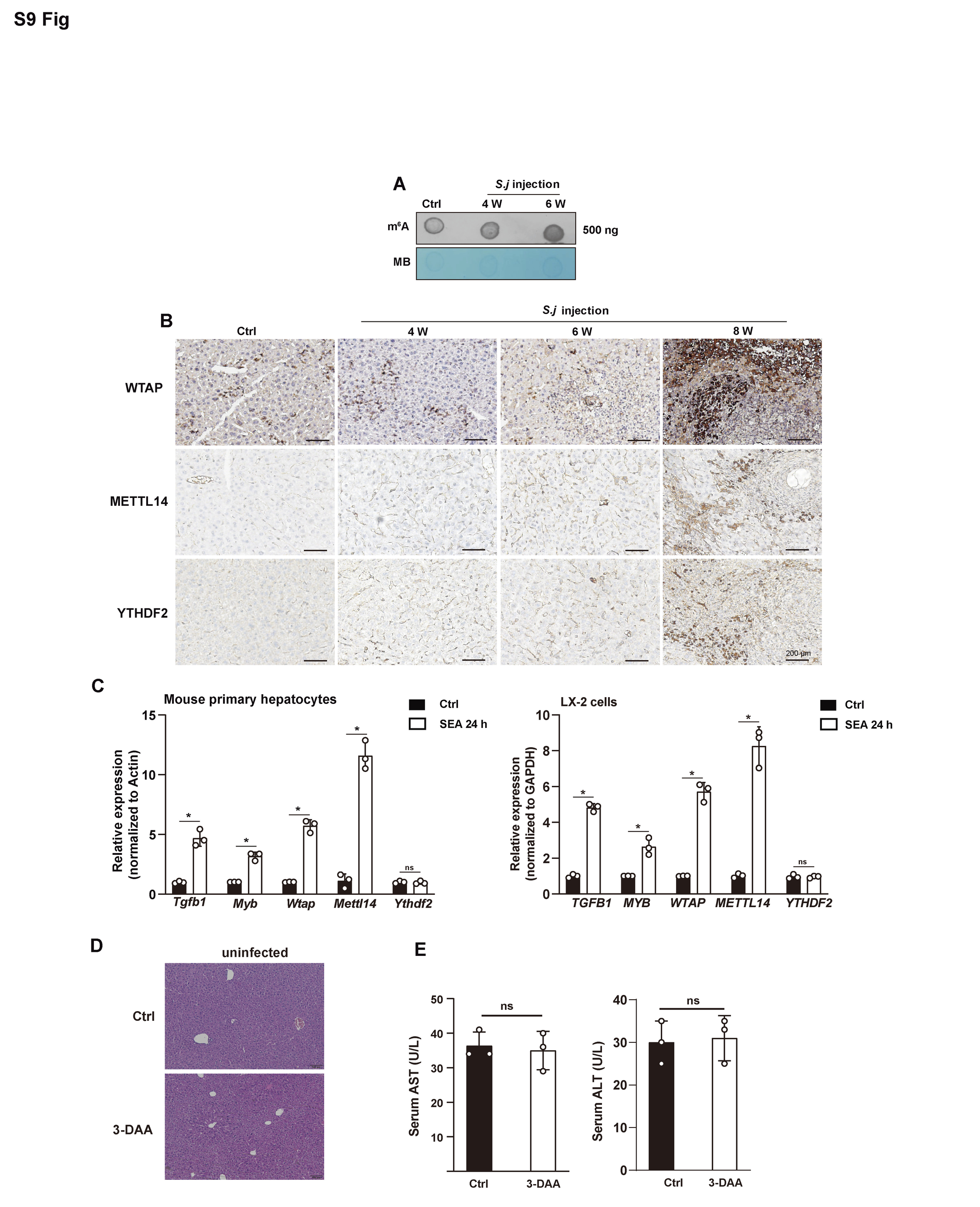

Supplement: S9 Fig — (A) Dot blot was used to measure m6A RNA modification in the livers of S. japonicum-infected mice. (B) Immunohistochemical (IHC) analysis was performed to evaluate WTAP, METTL14, and YTHDF2 expression in fibrotic liver tissues from mice infected with S. japonicum. (Scale bar: 200 μm). (C) Mouse primary hepatocytes and LX-2 cells were treated with SEA at concentrations of 60 μg/mL and 30 μg/mL, respectively, for 24 h, followed by real-time PCR analysis for TGFB1, MYB, WTAP, METTL14, and YTHDF2 mRNA. (D) H&E staining of liver sections from mice induced by 3-DAA (1.5 μg/g) (scale bar: 100 μm). (E) Plasma ALT and AST levels in mice. Data represent mean ± SD from three independent experiments. Statistical analyses were performed using 2-tailed Student’s t-test. *P < 0.05 versus ctrl. ns, not significant. (TIF) [file ppat.1014007.s009.tif]
