# Supplementary material for: Tristetraprolin attenuates schistosomiasis-induced liver fibrosis through m⁶A-mediated regulation of TGF-β1 mRNA stability
Source: PLoS Pathog. 2026 May 13;22(5):e1014007. doi: 10.1371/journal.ppat.1014007 (PMC13189414; doi:10.1371/journal.ppat.1014007)
Supplement: S1 Table — (DOCX) [file ppat.1014007.s010.docx]

S1 Table. Primers and shRNA sequences

Gene Symbol Sense Primer(5’-3’) Antisense Primer(5’-3’)

Primers for real-time PCR

*GAPDH* ACCATCTTCCAGGAGCGAGATC TGATGACCCTTTTGGCTCCCC

*TTP* TCGGGACCCTGGAGCCTGAG AGCCAGCGGTGCGAAGCC

*TGFB1* AGCTGTACCAGAAATACAGCA ATAACCACTCTGGCGAGTC

*MYB* GAAAGCGTCACTTGGGGAAAA TGTTCGATTCGGGAGATAATTGG

*α-SMA*  CAGGGCTGTTTTCCCATCCAT GCCATGTTCTATCGGGTACTTC

*COL1A1* CTGGAAGAGTGGAGAGTACTG GTCTCCATGTTGCAGAAGAC

*SMAD7* GGACAGCTCAATTCGGACAAC GTACACCCACACACCATCCAC

*WTAP* TGGCGAAGTGTCGAATGCTTA CAACTGCTGGCGTGTCTCCTT

*METTL14*  TGGACTTGGGATGATATTATGA CCCATTTTCGTAAACACACTCT

*YTHDF2* TAGCCAACTGCGACACATTC CACGACCTTGACGTTCCTTT

*TIMP1*  AACAGCCTGAGCTTAGCTC AAACAGGGAAACACTGTGC

*COL4A1*  CAGGTATTCCTGGGTTTGAC GGAAATCCTCTTGGACCAG

m*-Actin* GGCTGTATTCCCCTCCATCG CCAGTTGGTAACAATGCCATGT

m*-Ttp* GGAGGACTTTGGAACATAA GATGGAGTCCGAGTTTATGT

m-*Acta2* CGCTGCTCCAGCTATGTGTGA TTTGGCCCATTCCAACCATTAC

m*-Col1a1*  GCACGAGTCA CACCGGAAC CCAATGTCCAAGGGAGCCAC

m*-Col3a1* TGGTCCTCAGGGTGTAAAGG GTCCAGCATCACCTTTTGGT

m-*Il6* ACAACCACGGCCTTCCCTACTT CAGGATTTCCCAGCGAACATGTG

m- *Il8* CAGAAAGGAAGTGATAGCAGTCCCA CAAAGTGTCTAGAGGTCTCCCGAA

m-*Ccl2*  GGCTCAGCCAGATGCAGTTAA CTTGGTGACAAAAACACAGCTTC

m-*Ifng*  GGAGGAACTGGCAAAAGGATG GACCTGTGGGTTGTTGACCT

m-*Tnf* CCCTCACACTCAGATCATCT TGTCTTTGAGATCCATGCCG

m- *Il4* GGTCTCAACCCCCAGCTAGT GCCGATGATCTCTCTCAAGTGAT

m- *Il13*  CCTGGCTCTTGCTTGCCTT GGTCTTGTGTGATGTTGCTCA

m- *Il17a* GGAGAGCTTCATCTGTGTCTCTG TTGGCCTCAGTGTTTGGACA

m-*TGFB1* ACTGGAGTTGTACGGCAGTG GGGGCTGATCCCGTTGATT

m-*Mettl14* GACTGGCATCACTGCGAATG AGGTCCAATCCTTCCCCAGA

m-*Wtap*  TGCAAGAGTGCACCACTCAA AGCATTCGACACTTCGCCAT

m-*Ythdf2*  CAGGCAAGGCCGAATAATGC TCTCCGTTGCTCAGTTGTCC

m-*Myb* CAGCAGGCATTACCAACACAG TCTAGTCCCATGGCCCTCTC

Sequences for shRNAs

Ctrl TTCTCCGAACGTGTCACGTT

*TTP* shRNA 1 CCAGAGCATCAGCTTCTCGAGAAGCT

*TTP* shRNA 2 TACAAGACTGAGCTATGTCTCGAGACA

*WTAP* shRNA 1 CAAGAGATGAGTTCTCGAGAATTAAC

*WTAP* shRNA 2 TGGCAAGAGATGAGTTACTCGAGTAAC

*SMAD2* shRNA GCCTGATCTTCACAGTCATCA

*SMAD3* shRNA CTGTGTGAGTTCGCCTTCAAT

m*-Ttp* shRNA ACCACCTCCTCTCGATACAAGCTCGAGC

Primers for RIP-PCR / MeRIP-PCR

*MYB* CDS contains m6A site CCTCCTGGACAGAAGAGGAAG GTTCGTCCAGGCAGTAGCTT

*TGF-β1 5*'UTR contains m6A site CTCAGGCGCCCCCATTCC TGGGGAAAAGTCTTTGCGGG

Primers for ChIP-PCR

*WTAP* promoter (-500- 0) TCCAGACCGATCTGATTCACTG GCTTTATCAAACCTGTTCATCCC

*METTL14* promoter (-500- 0) GACAAAACTGAGGCTCACGAA CCAGGATAGCAGGTTCCCTT

*YTHDF2* promoter (-500- 0) CGGCTAACAGAGGATGACCAA TCTGGCTTCAGAAGAACGACAA

Primers for plasmid construction

pHAGE-*TTP* gtcgacATGGATCTGACTGCCATCTACGAG ctcgagTCACTCAGAAACAGAGATGCGATTG

pHAGE-*TTP-N-Zn -*FLAG ggatccATGGATCTGACTGCCATCTACGAGAG ctcgagTCACAGGTCTTCGCTAGGGTTG

pHAGE-*TTP-Zn-C-*FLAG gtcgacATGTACAAGACTGAGCTATGTCGG ctcgagTCACTCAGAAACAGAGATGCGATTG

pHAGE-*TTP-N -*FLAG ggatccATGGATCTGACTGCCATCTACGAGAG ctcgagTCAGCGCGAGGGGGTGGT

pHAGE-*TTP-Zn -*FLAG ggatccATGTACAAGACTGAGCTATGTCGGAC ctcgagTCATGGTGGTGGTGAGGTCC

pHAGE-*TTP-C-*FLAG gtcgacATGCCAGGCCTGGCCGGCCCTTC ctcgagTCACTCAGAAACAGAGATGCGATTG

pHAGE-*Smad2/3-MH1-*HA ggatccATGCCGCCAGTTGTGAAGAGACTGC ctcgagTCATGTCTCAACTCTCTGATAGTGGT

pHAGE-*Smad2/3-MH2-*HA ggatccATGACTTACTCAGAACCTGCATTTTGG ctcgagTCACATCTGAGTTAATACTTTGTCCAACC

PGL *WTAP* actcgtTTGCTTGAGCCCAGGAAGTCA aagcttGTGGAGTAGACTCCCTGAAGTAAGA

PGL *METTL14* gagctcGACGCCATTCACTATGGAGAAATA aagcttACTTCTCCTTTATCTCTCTTTCCCA

PGL *YTHDF2* acgcgtAACTGGGAACTTCTGGCAAAC ctcgagTAAAGCAGAGCGCCGAACA

pMIR TGF-β1 5'UTR contains m6A site gagctcGAAAAAAACTTTTGAGACTTTTCCG ctcgagGGAAAAGTCTTTGCGGGAGG
