# Supplementary material for: Tristetraprolin attenuates schistosomiasis-induced liver fibrosis through m⁶A-mediated regulation of TGF-β1 mRNA stability
Source: PLoS Pathog. 2026 May 13;22(5):e1014007. doi: 10.1371/journal.ppat.1014007 (PMC13189414; doi:10.1371/journal.ppat.1014007)
Supplement: S2 Table — (DOCX) [file ppat.1014007.s011.docx]

S2 Table. The information of primary antibodies

| **Antibodies** | **Source** | **Identifier** |
| --- | --- | --- |
| Rabbit Control IgG | ABclonal | Cat#AC005 |
| HRP-conjugated Mouse-IgG Polyclonal antibody | Proteintech | Cat#HRP-10283 |
| Mouse monoclonal Anti-GAPDH | Proteintech | Cat#60004-1-Ig |
| Mouse monoclonal Anti-α-Tubulin | Proteintech | Cat#66031-1-Ig |
| Rabbit Polyclonal anti-α-SMA | Proteintech | Cat#14395-1-AP |
| Mouse monoclonal anti-COL1A1 | Proteintech | Cat#67288-1-Ig |
| Mouse monoclonal anti-WTAP | Proteintech | Cat#60188-1-Ig |
| Rabbit Polyclonal anti-YTHDF2 | Proteintech | Cat#24744-1-AP |
| Rabbit Polyclonal anti-METTL14 | Proteintech | Cat#26158-1-AP |
| Rabbit Polyclonal anti-TTP | Millipore | Cat#ABE285 |
| Rabbit Polyclonal anti-m6A | Epigentek | Cat#A-1801-020 |
| HRP horseradish peroxidase | Proteintech | Cat#PR30015 |
| Monoclonal antibody anti-FLAG | Proteintech | Cat#66008-4-Ig |
| Human/mouse SMAD2/3 antibody | R&D Systems | Cat#AF3797 |
