# Supplementary material for: Tristetraprolin attenuates schistosomiasis-induced liver fibrosis through m⁶A-mediated regulation of TGF-β1 mRNA stability
Source: PLoS Pathog. 2026 May 13;22(5):e1014007. doi: 10.1371/journal.ppat.1014007 (PMC13189414; doi:10.1371/journal.ppat.1014007)
Supplement: Attachment — Submitted filename: Point-by-Point Response to Reviewers.pdf [file ppat.1014007.s013.pdf]

# Point-by-Point Response to Reviewers

**Responses to comments of the Reviewer #1**

**Reviewer #1:**
In this study, the authors investigated the potential role of Tristetraprolin (TTP) in the pathogenesis of *Schistosoma japonicum*-induced liver fibrosis. Using a murine model, they first demonstrated that TTP expression is upregulated at both the RNA and protein levels in the liver following infection. Notably, overexpression of TTP ameliorated infection-associated liver fibrosis. To further elucidate the underlying mechanisms, the authors employed various cell lines and found that TTP negatively regulates the mRNA stability of TGF-β1 and MYB by promoting N6-methyladenosine (m6A) RNA methylation, thereby inhibiting hepatic stellate cell activation. Additionally, TTP was shown to activate transcription of WT1-associated protein (WTAP) through its interaction with SMAD2/3. Furthermore, using an m6A RNA methylation inhibitor, the study demonstrated that TTP protects against *S. japonicum*-induced liver fibrosis in vivo by enhancing m6A RNA methylation. These findings reveal novel mechanisms involving TTP-mediated m6A RNA hypermodification in the pathogenesis of S. japonicum-induced liver fibrosis, with potential implications for the development of future therapeutic strategies. Overall, the experiments were well-performed, making the study of interest to the journal's readership. However, the manuscript suffers from a general lack of clarity, as detailed below, which needs to be addressed.

**We sincerely thank you for your positive comments on our manuscript. We appreciate the thoughtful review you provided and have made every effort to provide a satisfactory response to your suggestions.**

Major Issues:
1. Important modifications are required, as detailed below in Part III.
**Response: Thank you so much for your suggestions. We have attempted to address your comments satisfactory in Part III.**

Minor Issues:
1. Many sentences and statements throughout the manuscript are confusing or ambiguous and require revision for clarity. For example:
• Lines 41–42: "TTP is upregulated in *S. japonicum*-induced liver fibrosis" — it is unclear in which tissue this upregulation occurs. Please specify.
**Response: Thank you. The original sentence "TTP is upregulated in *S. japonicum*-induced liver fibrosis" has been revised to "TTP is upregulated in the liver during *S. japonicum*-induced liver fibrosis" (Page 3, lines 44-45)**

• Lines 46–47: "TTP activates WATP transcript" — do the authors mean that TTP activates WTAP mRNA molecule or transcription of the WTAP gene? This should be clearly stated.

**Response: Thank you for your feedback. The original sentence “TTP activates WATP transcript” has been revised to “TTP activates the transcript *WTAP* gene” (Page 3, lines 49-50)**

• Lines 59–60: “the mechanism underlying the fine-tuning regulation of inflammatory cytokines and chemokines” — it is unclear whether this refers to regulation of their gene expression or their biological function.

**Response: Thank you for pointing out this ambiguity. We clarify that "the mechanism underlying the fine-tuning regulation of inflammatory cytokines and chemokines" specifically refers to the regulation of their gene expression. Accordingly, we have revised the original phrase to "the mechanism governing the fine-tuning regulation of inflammatory cytokine and chemokine gene expression" to eliminate any potential confusion (Page 5, lines 62-64).**

• The authors are strongly encouraged to thoroughly revise the manuscript for clarity, ideally with assistance from a native English speaker or professional language editing service.

**Response: Thank you for your suggestion. We have thoroughly revised the manuscript to enhance clarity and have also engaged a professional language editing service to refine the text.**

2. The description of the results in Figure 1A should clearly indicate that TTP expression is being assessed at the protein level. Additionally, in lines 129–133, the current wording implies that TTP, rather than albumin, is predominantly located in hepatocytes. This should be corrected to accurately reflect that albumin is the hepatocyte marker, and TTP's localization is being evaluated in relation to it in hepatocytes, as well as in HSCs.

**Response: Thank you for pointing out these important issues. We have revised the description of Figure 1A to explicitly clarify that TTP expression is assessed at the protein level (new Fig. 1A). For lines 129–133, we have revised the wording to clarify that albumin acts as a hepatocyte marker. Moreover, we have further clarified that we are assessing TTP’s localization relative to this marker within hepatocytes, as well as its expression in hepatic stellate cells (HSCs) (new Fig. 1C). In addition, we isolated hepatocytes (HCs), hepatic stellate cells (HSCs), and macrophages from the livers of normal mice. We found that the mRNA level of TTP is significantly higher in primary HCs and HSCs compared to macrophages (new Fig. S1D). The revised content has been carefully double-checked to ensure accuracy and preclude any potential misinterpretation (Page 9, lines 143-144; Page 9, lines 145-147; Page 10, lines 148-153; Page 28, lines 536; Page 29, lines 537-545; Page 44, lines 933-934; Page 45, lines 946-951; Page 51, lines 1078-1079).**

3. The result descriptions for Figures 2 and 3 should include comparisons between TTP knockdown or overexpression and the corresponding controls from non-infected

animals. Currently, the descriptions focus primarily on infected animals, which limits the interpretation of TTP's specific role in infection-induced changes. Including these comparisons would provide a more complete understanding of TTP's effects under both baseline and infected conditions.

**Response: Thank you for this valuable suggestion. In accordance with your recommendations, TTP knockdown and overexpression were confirmed in the respective TTP knockdown, overexpression, and control groups derived from non-infected animals via qRT-PCR and Western blot analyses (new Figs. S2A and S4A). We have included these new experimental data in the revised manuscript (New Figs. S2A and S4A; Page 10, lines 166-168; Page 12, lines 204-205; Page 52, lines 1087-1089; Page 53, lines 1109-111).**

4. In Figure 6A, it would be important to clarify whether TTP can directly interact with WTAP. Was WTAP identified in the mass spectrometry (MS) data? If so, this should be explicitly stated. If WTAP was not detected in the MS results, this negative finding is also worth mentioning, as it may suggest that the interaction is indirect or occurs under specific conditions not captured in the assay.

**Response: Thank you for this insightful comment. Our results showed that WTAP was not detected in the mass spectrometry (MS) data (RIDE database: PXD056357). Moreover, our previous coimmunoprecipitation (Co-IP) data indicated that TTP does not interact with WTAP in hepatocytes (DOI: 10.1172/jci.insight.149276). Taken together, these findings demonstrate that TTP does not interact with WTAP. Detailed information has been incorporated into the results section (Page 17, lines 303-305).**

5. Several other proteins identified in the list may also function as transcriptional activators. Therefore, the authors should provide a clear rationale for selecting SMAD2/3 for further analysis. Explaining this choice—whether based on prior literature, expression patterns, relevance to liver fibrosis, or preliminary data—would strengthen the interpretation of the results and the logic behind the experimental design.

- **Response: Thank you for this valuable suggestion. Extensive prior literature demonstrated that SMAD2/3 are core transcriptional regulators in the TGF-β signaling pathway, which is critically involved in the pathogenesis of *S. japonicum*-induced liver fibrosis (DOI: 10.1371/journal.ppat.1012153, DOI: 10.1038/Nature25784). Moreover, previous reports have shown that SMAD2/3 expression is significantly upregulated in *S. japonicum*-induced liver fibrosis tissues, which is consistent with TTP's expression pattern (DOI: 10.1186/s13071-022-05584-1). We have revised the manuscript to incorporate this detailed explanation into the results section (Page 17, lines 306-308; Page 41, lines 879-882; Page 42, lines 883-886).**

6. It is unclear whether the DAA inhibitor was administered as a single intraperitoneal injection at 42 days post-infection. If so, the authors should clarify the duration of DAA's

activity in vivo, as it is questionable whether a single dose would remain effective for an extended period. If possible, additional details regarding the dosing regimen, half-life, and pharmacokinetics of DAA in this model would be helpful for evaluating the validity of the experimental approach.

**Response: Thank you for this valuable suggestion. Previous studies have shown that schistosome eggs start to deposit in the liver approximately 42 days after infection, triggering significant hepatic granuloma formation and initiating HSC activation (DOI: 10.1038/s41598-017-02422-8, DOI: 10.3389/fimmu.2020.00795, DOI: 10.1186/s13071-022-05584-1). By 42 days post-infection, the fibrotic process is well-established, rendering this an optimal window to assess the effect of 3-DAA. The dose of 3-DAA used (1.5 μg/g) was selected based on previous reports showing its efficacy in inhibiting $m^6A$ methylation without obvious toxicity in murine models（DOI: 10.1172/jci.insight.149276）. Additionally, our dot blot assay revealed that 3-DAA treatment (1.5 μg/g, once every two days) dramatically attenuated the global $m^6A$ levels in the livers of mice (Fig. 8B). Moreover, *in vivo*, we monitored mice's liver function (ALT/AST levels) and histopathological changes during treatment, and no signs of systemic toxicity were observed in 3-DAA-injected mice. These data are included as new Fig. S9D-E and detailed in the Results section (Page 21, lines 395-397， lines399-401; Page 22, lines 402-405, Page 42, lines 887-889; Page 55, lines 1170-1071).**

7. In lines 356–357, the text incorrectly refers to SMAD2/3 as an important m6A writer. This should be corrected, as it is WTAP, not SMAD2/3, that functions as a core component of the m6A methyltransferase complex. Please revise this statement to accurately reflect the roles of these proteins.

**Response: Thank you for your feedback. We have implemented the correction according to your advice (Page 23, lines 417-419).**

8. Including a schematic diagram or cartoon summarizing the proposed pathway would greatly enhance the manuscript's clarity and impact. For example, a visual representation illustrating the sequence: infection → TTP induction → interaction with SMAD2/3 to increase WTAP transcription → enhanced m6A methylation → regulation of TGF-β and MYB mRNA stability → hepatic stellate cell (HSC) activation → liver fibrosis would help readers better understand the complex molecular mechanisms described.

**Response: Thank you for this insightful suggestion. As requested, we have added the schematic diagram summarizing the proposed molecular pathway as Fig. 9 in the revised manuscript (Page 51, lines 1067-1072).**

**<u>Responses to comments of the Reviewer #2</u>**

**Reviewer #2:**

-Well written and easy to read.

**Response: Thank you for your suggestion. We have thoroughly revised the manuscript to enhance clarity and have also engaged a professional language editing service to refine the text.**

-The quality of all figures was very low (blurry). Fonts small and fuzzy/blurry. Hard to read/see in many instances and appreciate the data. The blurry/ fuzziness was not an issue in the supplemental file, so that might be an issue with the Pdf file for the main text and should be corrected.

**Response: Thank you for this insightful feedback. All figures have been reuploaded in high resolution to address the concerns.**

-In introduction, there is no information and background on the importance of TGF-β1 and MYB in schistosome/schistosome induced fibrosis and whether there is any previous literature. This is important since the manuscript is focused on the regulation of these molecules.

**Response: Thank you for this critical feedback. The background on the importance of TGF-β1 and MYB in schistosome-induced fibrosis has been added to the Introduction section of the revised manuscript (Page 6, lines 87-93; Page 40, lines 843-851).**

-Figure legends: No information regarding how many times experiments have been performed, biological repeats and technical repeats. This is the problem for all figures in the main text and supplementary figures.

**Response: Thank you for pointing out this critical issue. According to previous reports (DOI: 10.1038/s41586-021-03313-9), we have revised all figure legends—both in the main text and supplementary materials—to clearly specify the number of experimental repeats, biological replicates, and technical replicates. Additionally, we have incorporated this information into the Materials and Methods section and figure legends of the revised manuscript (Page 34, lines 659-662; Page 35, lines 663-666; Page 45, lines 952-954; Page 46, lines 970-972; Page 47, lines 989-991; Page 48, lines 1006-1007; Page 48, lines 1019-1021; Page 50, lines 1046-1048, 1062-1064; Page 51, lines 1081-1082; Page 52, lines 1095-1096, lines 1104-1105; Page 53, lines 1117-1118,lines 1126-1127; ; Page 54, lines 1137-1138, lines 1149-1151; ; Page 55, lines 1159-1161,lines 1172-1173).**

-One concerning issue is that, while many pathways discussed could be relevant and compelling in the broader context of liver fibrosis, their applicability weakens when the authors attempt to connect them specifically to schistosomiasis-related liver fibrosis (see details in Part II).

**Response: Thank you so much for your suggestions. As noted, the TTP identified in our study alleviates schistosomiasis-induced liver fibrosis through m$^6$A-mediated regulation of TGF-β1 mRNA stability, and this mechanism is not necessarily restricted to schistosomiasis-induced fibrosis alone. Whether this signaling pathway is conserved across other fibrosis types warrants further**

**investigation. We have discussed his point in the revised manuscript (Page 26, lines 495-497; Page 27, lines 498-503).**

-Authors do not discuss the cellular source of TTP anywhere in the manuscript and what cells they think might be the source in the context of schistosome in the liver.
**Response: Thank you for pointing out this critical issue. Our double immunofluorescence staining of liver sections demonstrated that TTP's localization relative to this marker within hepatocytes, as well as its expression in hepatic stellate cells (HSCs) (new Fig 1C). In addition, we isolated hepatocytes (HCs), hepatic stellate cells (HSCs), and macrophages from the livers of normal mice. We found that the mRNA level of TTP is significantly higher in primary HCs and HSCs compared to macrophages (new Fig S1D). Moreover, hepatic TTP knockdown significantly promoted *S. japonicum*-induced liver fibrosis *in viv*o. Collectively, these results imply that TTP expressed in hepatocytes mediates the paracrine secretion of TGF-β, thereby inducing HSC activation. We have added more detailed information in the results and discussion section (Page 9, lines 143-144; Page 9, lines 145-147; Page 10, lines 148-153; Page 28, lines 536; Page 29, lines 537-545; Page 44, lines 933-934; Page 45, lines 946-951)**

Major Issues:
Reviewer #2:
For in vivo experiments in figures 2 and 3 weights of infected livers should be included. Also weights of uninfected livers as control should be included.
**Response: Thank you for this constructive suggestion. As suggested, liver weight proportion data from *S. japonicum*-infected and uninfected animals after AAV injection have been incorporated into new Fig. 2E and 3E (Page 11, lines 182-184; Page 12, lines 209-211; Page 46, lines 967-968; Page 47, lines 986-987).**

Fig 2D and Fig. 3D and Fig. 8 show the granuloma size which is significant, but it is not enough. Authors need to show the total numbers of eggs in liver tissue (per gr or per field) as well to make sure that the parasite load was the same.
**Response: Thank you for your insightful comment. Based on previous studies (DOI: 10.1371/journal.ppat.1010233), we measured the egg loads in the livers of TTP knockdown, overexpression, and control groups. The results showed no significant differences in hepatic egg loads among the three groups, suggesting that alterations in TTP expression do not affect egg deposition in the liver following *S. japonicum* infection (new Fig. 2D and 3D; Page 11, lines 178-180; Page 12, lines 208-209; Page 46, lines 964-967; Page 47, lines 983-986).**

-Line 178: authors state "To systematically elucidate the role of TTP in liver fibrosis, we reanalyzed our previously published RNA-Seq dataset from TTP-knockdown cells (GSE157581)". The study that they are referring to is acute liver failure and is different from liver fibrosis associated with schistosomiasis. They found these 2 molecules TGF-β1 and MYB from this RNA-Seq dataset and they validated using qPCR in hepatocytes

and LX-2 cells but none were performed in cells stimulated with soluble egg antigens (SEA) or schistsome eggs, making it relevant to liver fibrosis associated with schistosome infection.

**Response: Thank you for this valuable suggestion. The GSE157581 dataset comprises the RNA-Seq analyses of TTP-knockdown cell lines and is not associated with acute liver injury. In response to your suggestion, we measured the mRNA expression of TTP, TGF-β1, and MYB in hepatocytes and LX-2 cells stimulated with soluble egg antigens (SEA). Our new data indicate that SEA stimulation significantly increased the expression of these three molecules in both cell types. This result has been incorporated into the updated supplementary figure (New Figs. S1E and S9C; Page 10, lines 157-158; Page 21, lines 386-390; Page 28, lines 533-535; Page 43, lines 930-932; Page 55, lines 1167-1170).**

-Similar to the comment above, experiments in Figures 6 and 7 are interesting per say but none are performed in the context of schisto, which could have been done in the presence of eggs or SEA. For example, the authors show that TGF-β treatment significantly increased the mRNA expression of WTAP and METTL14 at the mRNA level (Fig. S3G), but what does that mean in the context of schisto infection and/or upon egg stimulation in vitro which is the focus of this manuscript?

**Response: Thank you for this valuable suggestion. In response to your comment, we measured the mRNA expression of WTAP and METTL14 in hepatocytes and LX-2 cells stimulated with soluble egg antigens (SEA). Our new data indicate that SEA stimulation significantly increases the expression of these two molecules in both cell types, and this result has been incorporated into the updated supplementary figure (New Fig. S9C). Additionally, the protein expression levels of WTAP and METTL14 have been included in Figure 9B. Collectively, these findings confirm that *S. japonicum* infection upregulates the expression of WTAP and METTL14, reinforcing their relevance to the pathological process of schistosomiasis (Page 21, lines 386-391; Page 28, lines 533-535; Page 43, lines 930-932; Page 55, lines 1167-1170).**

-Line 65: "our study has revealed TTP alleviates schistosomiasis-induced liver fibrosis by regulating TGF-β1 and MYB mRNA stability in an m6A-dependent manner." For reasons mentioned above this sentence needs to be corrected

**Response: Thank you for pointing out this issues. Our *in vitro* data reveal TTP negatively regulate mRNA stabilization of TGF-β1 and MYB by activating WTAP transcription. Notably, using a hepatocyte and HSC co-culture system, TTP suppresses HSC activation by negatively regulating TGF-β1 expression. Moreover, using an N6-methyladenosine ($m^6A$) RNA methylation inhibitor, we confirmed that $m^6A$ RNA methylation contributes to TTP-mediated protection against schistosomiasis-induced liver fibrosis. Taken together, our data suggest that TTP alleviates schistosome-induced liver fibrosis through $m^6A$-mediated regulation of TGF-β1 mRNA stability. Per your valuable suggestion, we have**

**revised the sentences of our manuscript to "our study has revealed TTP alleviates schistosomiasis-induced liver fibrosis by regulating TGF-β1 mRNA stability in an m$^6$A-dependent manner" (Page 5, lines 68-70).**

-Fig. 8 shows that m6A RNA methylation contributes to TTP-mediated protection against liver fibrosis but it does not show that it is via regulating TGF-β1 and MYB stability, and the title of the manuscript "TTP alleviates schistosomiasis-induced liver fibrosis by regulating TGF-β1 and MYB stability in an m6A-dependent manner" needs to be corrected.

**Response: Thank you for your insightful comment. We agree with your comment that we have not formally demonstrated, in animal models, that TTP protects against schistosome-induced liver fibrosis through regulating the mRNA stability of TGF-β1 and MYB in an m$^6$A-dependent manner. However, our *in vitro* data reveal TTP negatively regulate mRNA stabilization of TGF-β1 and MYB by activating *WTAP* transcription. Notably, using a hepatocyte and HSC co-culture system, TTP suppresses HSC activation by negatively regulating TGF-β1 expression. Moreover, using an N6-methyladenosine (m$^6$A) RNA methylation inhibitor, we confirmed that m$^6$A RNA methylation contributes to TTP-mediated protection against schistosomiasis-induced liver fibrosis. Taken together, our data suggest that TTP alleviates schistosome-induced liver fibrosis through m$^6$A-mediated regulation of TGF-β1 mRNA stability. Per your valuable suggestion, we have revised the title of our manuscript to "TTP alleviates schistosome-induced liver fibrosis via m$^6$A-mediated regulation of TGF-β1 mRNA stability". To ensure the academic accuracy of the manuscript, we have revised the relevant descriptions accordingly (Page 1, lines 1-3; Page 5, lines 68-70).**

- Confocal image in Fig. 1C is not convincing. Fig. 1C: middle a-SMA (there is a lot of green that is not merged with red). I see more colocalization with AJB (top) than a-SMA-Bottom. Qualification could be very helpful. There is also staining for F480 but there is no explanation in the text regarding F480 staining and why they included it.

**Response: Thank you for your valuable feedback. To ensure unbiased and representative quantification, we reanalyzed our confocal images by utilizing multiple images derived from distinct tissue sections, following the methods described in a published manuscript (DOI: 10.1038/s41467-023-43743-9) (new Fig. 1C). The explanation of F4/80 (a specific marker for macrophages) has been included in the revised version (Page 9, lines 145-147; Page 10, lines 148-149; Page 45, lines 946-951).**

- Authors show that TTP modulates HSC activation by regulating TGF-β1 expression (Fig. S4A). How about a role for MYB? Since it is also included in the title of the manuscript? Did the authors investigate the role for this molecule? Any previous literature regarding this? It is important that authors discuss this.

**Response: Thank you for your valuable feedback. We agree with your comment**

**that we have not formally demonstrated, in animal models, that TTP protects against schistosome-induced liver fibrosis through regulating the mRNA stability of TGF-β1 and MYB in an $m^6A$-dependent manner. However, our *in vitro* data reveal TTP negatively regulate mRNA stabilization of TGF-β1 and MYB by activating WTAP transcription. It has been reported that MYB promotes hepatic fibrosis by activating the transcription of profibrotic genes in HSCs (DOI: 10.1002/hep.31188). Following your valuable suggestion, we have revised the manuscript title to "TTP alleviates schistosome-induced liver fibrosis via $m^6A$-mediated regulation of TGF-β1 mRNA stability" and incorporated background information on MYB in the Introduction section. To ensure the academic accuracy of the manuscript, we have carefully revised the relevant descriptions accordingly (Page 1, lines 1-3; Page 6, lines 92-93; Page 40, lines 848-851).**

Minor Issues:

Line 87: Authors mention that TTP negatively regulates several proinflammatory cytokines such as IL-6, IL-8 and CCL2. Since these proinflammatory molecules have an important role in schistosomiasis, it makes sense that authors would investigate whether TTP also affects these cytokines and chemokines during schistosome infection.

**Response: We thank the reviewer for highlighting this important point. We agree that proinflammatory cytokines such as IL-6, IL-8, and CCL2 play critical roles in schistosomiasis pathogenesis—particularly in regulating granulomatous inflammation and subsequent liver fibrosis (DOI: 10.1111/cmi.12316; DOI: 10.1093/infdis/jis524). Per your suggestion, we measured IL-6, IL-8, and CCL2 mRNA expression in TTP-knockdown mice after *S. japonicum* infection. Our new data indicated TTP knockdown significantly up-regulated the mRNA expression of IL-6, IL-8, and CCL2 in *S. japonicum*-induced murine model (New Fig. S3A). Additionally, according to the previously studies, TTP negatively regulates CCL2, IL-6, and IL-8 expression though binding to its 3'UTR in the ARE regions (DOI: 10.1093/nar/gkw474, DOI: 10.2741/3920). Our study focused on investigating the mechanism by which TTP regulates TGF-β1 mRNA stability via $m^6A$ methylation through its interaction with SMAD2/3. The involvement of TTP in $m^6A$-mediated TGF-β1 mRNA degradation provides an additional regulatory layer in the pathogenesis of *S. japonicum*-induced liver fibrosis. We have added more detailed information in the results and discussion section (Page 12, lines 192-197; Page 26, lines 495-497; Page 27, lines 498-501).**

-This is a minor comment and would make the manuscript stronger if included: How about the cytokine profile of the liver of these mice? Would be a great addition to the manuscript to assess a few Th1 and Th2 and Th17 cytokines.

**Response: Thank you for this valuable suggestion. We have performed additional real-time PCR assays to quantify the mRNA expression levels of core Th1 (IFN-γ, TNF-α), Th2 (IL-4, IL-13), and Th17 (IL-17A) cytokines. Our new results**

**have been included in the revised manuscript (New Fig. S3B-S3D; Page 12, lines 197-199; Page 52, lines 1101-1104).**

-Figure 5: Authors already have reported that TTP modulates m6A modification by regulating the expression of WTAP and in this study (Fig 5) they just investigate whether WTAP was involved in the regulation of TGF-β1 and MYB. I don't find this novel and can be moved to supplemental. This is a minor comment.

**Response: Thank you for your suggestion. Figures 5A-D demonstrate that WTAP promotes the degradation of TGF-β1 and MYB mRNA via regulation of m$^6$A RNA methylation. Additionally, Figure 5E indicates that TTP enhances m$^6$A-mediated post-transcriptional regulation of TGF-β1 and MYB by modulating WTAP. Given that these data delineate the molecular mechanism by which TTP negatively regulates TGF-β1 and MYB expression through modulation of WTAP, we consider it more appropriate to include Figure 5 in the main text. We hope you can understand. To more accurately summarize the findings of Figure 5, we have revised the relevant descriptions in the manuscript accordingly (Page 15, Lines 265-266; Page 16, lines 288-289).**

-Please explain in the text that a-SMA is marker for activated HSC

**Response: We agree with your suggestion. Numerous studies have established that α-smooth muscle actin (α-SMA) is a well-recognized specific marker for activated hepatic stellate cells (HSCs) (DOI: 10.1016/j.cell.2019.07.021). We have supplemented detailed explanatory content regarding this marker in the Results section of the revised manuscript (Page 9, lines145-147).**

-Line 137: Authors explain data in the text about Fig. 1D which does not exist-Please correct

**Response: Thank you for pointing out this error. We have corrected Line 155-156 by revising the figure citation to the correct one (Fig. 1D)**

-Line 203: "Wild" and not "Wile". Please correct

**Response: Thank you for your feedback. We have implemented the correction according to your advice in line 247.**

-Line 339: why the injection of DAA, was performed at 42 days after S. japonicum infection? Would be great if authors describe the rationale or refer to previous literature if any.

**Response: Thank you for this valuable suggestion. Previous studies have shown that schistosome eggs start to deposit in the liver approximately 42 days after infection, triggering significant hepatic granuloma formation and initiating HSC activation (DOI: 10.1038/s41598-017-02422-8, DOI: 10.3389/fimmu.2020.00795, DOI: 10.1186/s13071-022-05584-1). By 42 days post-infection, the fibrotic process is well-established, rendering this an optimal**

**window to assess the effect of 3-DAA. These data are included as new Figs. S9D–E, with detailed descriptions provided in the Results section (Page 21, lines 395-401; Page 22, lines 402-405; Page 39, lines 887-889; Page 55, lines 1170-1172).**

-A problem of this work is that no dose response for the inhibitor 3-DAA is used and we don't know if it is causing cell death or toxicity.
**Response: The dose of 3-DAA used (1.5 μg/g) was selected based on previous reports showing its efficacy in inhibiting m$^6$A methylation without obvious toxicity in murine models（doi: 10.1172/jci.insight.149276）. Moreover, *in vivo*, we monitored mice liver function (ALT/AST levels), and histopathological changes during treatment, and no signs of systemic toxicity were observed in 3-DAA-injected mice. These data are included as new Figs. S9D–E, with detailed descriptions provided in the Results section (Page 21, lines 399-401; Page 22, line 405; Page 55, lines 1170-1172).**

**<u>Responses to comments of the Reviewer #3</u>**
**Reviewer #3:**
This study presents a comprehensive investigation into the role of Tristetraprolin (TTP) in Schistosoma japonicum-induced liver fibrosis, with a focus on its regulation of m6A RNA methylation through interactions with SMAD2/3 and subsequent effects on TGF-β1 and MYB expression. The topic is novel and highly relevant to the field of hepatology and parasitology. The experimental design is generally rigorous, incorporating in vivo models, multi-omics approaches, and detailed mechanistic insights. However, several critical issues pertaining to methodological justification, analytical quantification, and experimental controls need to be addressed to fully strengthen the validity and impact of the conclusions.
**Response: We sincerely thank you for your positive comments on our manuscript. We appreciate the thoughtful review you provided and have made every effort to provide a satisfactory response to your suggestions.**

Major Issues:
1. Quantification of Immunofluorescence (IF) Data: While Figure 1C suggests TTP is "predominantly localized in hepatocytes," the claim requires robust quantitative support. The authors should perform and report quantitative image analysis (e.g., Pearson's correlation coefficient for co-localization with cell-specific markers, or mean fluorescence intensity measurements across different cell types) to objectively substantiate this key observation. Additionally, quantifying TTP mRNA or protein levels in sorted populations of hepatocytes, hepatic stellate cells (HSCs), Kupffer cells, and endothelial cells from fibrotic livers would provide definitive evidence and greatly strengthen the foundation of the study.
**Response: Thank you for pointing out this critical issue. To ensure unbiased and representative quantification, we reanalyzed our confocal images using multiple images from distinct tissue sections, in accordance with the method detailed in**

**the new Fig. 1C (DOI: 10.1038/s41467-023-43743-9). This reanalysis included the calculation of Pearson's correlation coefficients between TTP and cell-specific markers (hepatocytes: albumin; HSCs: α-SMA; macrophages: F4/80) to quantify colocalization. In addition, we isolated hepatocytes (HCs), hepatic stellate cells (HSCs), and macrophages from the livers of normal mice. We found that the mRNA level of TTP is significantly higher in primary HCs and HSCs compared to macrophages (new Fig S1D; Page 9, lines 143-144; Page 9, lines 145-147; Page 10, lines 148-153; Page 28, lines 536; Page 29, lines 537-545; Page 44, lines 933-934; Page 45, lines 946-951; Page 51, lines 1078-1079)**

**.**

2. Rationale for Focus on Hepatocytes: The study focuses on hepatocytes as the primary functional site of TTP, despite also detecting TTP in HSCs (Fig. 1C). The rationale for prioritizing hepatocytes over HSCs is not adequately explained. Given that HSCs are the central effector cells in fibrosis, the authors should either: Provide functional data comparing TTP effects in hepatocytes vs. HSCs, or Clearly justify their focus on hepatocytes and discuss the potential paracrine mechanisms by which hepatocyte TTP influences HSC activation.

**Response: Thank you for this insightful comment. Our data found that the mRNA level of TTP is significantly higher in primary HCs and HSCs compared to macrophages (new Fig, S1D). Moreover, hepatic TTP knockdown significantly promoted *S. japonicum*-induced liver fibrosis *in viv*o. Collectively, these results imply that TTP expressed in hepatocytes mediates the paracrine secretion of TGF-β, thereby inducing HSC activation. We have added more detailed information in the results and discussion section (Page 9, lines 143-144; Page 9, lines 145-147; Page 10, lines 148-153; Page 19, lines 359; Page 26, lines 477-479; Page 28, lines 536; Page 29, lines 537-545; Page 44, lines 933-934; Page 45, lines 946-951; Page 51, lines 1078-1079)**

3. Justification for Experimental Timeline: The choice to administer AAV vectors at day 10 post-infection is a critical methodological detail that lacks justification. Please provide a scientific rationale for this specific time point.

- **Response: We appreciate the reviewer's critical comment regarding the experimental timeline. Previous studies indicated AAV administration at the early fibrotic stage (day 10–14 post-infection) achieves maximal transgene expression in hepatocytes and optimal therapeutic efficacy in schistosomiasis-induced fibrosis (DOI: 10.1073/pnas.1715965115). New relevant literatures have been added in the revised manuscript (Page 11, lines 169-170; Page 41, lines 876-878).**

4. In Vivo Control Group: The authors have convincingly demonstrated that TTP overexpression via AAV8 delivery ameliorates liver fibrosis. However, the design of the in vivo experiment lacks a necessary control group to fully validate the findings. The

current study includes only two groups: AAV8-Ctrl and AAV8-TTP. The AAV8-Ctrl group is intended to serve as the control, but it is not sufficient to rule out the potential effects of the AAV8 viral vector itself on the progression of schistosomiasis-induced liver fibrosis. It is well-known that viral vectors can sometimes elicit innate immune responses or other off-target effects that might inadvertently influence the disease model. Therefore, to unequivocally attribute the observed therapeutic effects solely to TTP overexpression, a third control group—a non-treatment group (e.g., injected with PBS or saline)—is essential. This would allow the authors to confirm that any difference between the AAV8-TTP and AAV8-Ctrl groups is specifically due to the action of TTP.

**Response: We appreciate the reviewer's insightful comment. As per your suggestion, we have added a non-treatment control group (injected with PBS) in the revised study to rule out potential off-target effects of the AAV vector itself on schistosomiasis-induced liver fibrosis. New H&E and Masson's trichrome staining results demonstrate that AAV-Ctrl injection did not affect the progression of schistosomiasis-induced liver fibrosis. These new data have been included in the revised manuscript (new Figs. S2B-E; Figs. S4B-E; Page 11, lines 172-175; Page 12, lines 206-208; Page 52, lines 1089-1095; Page 5, lines 1111-1116).**

5. Statistical Transparency: For all histological data (e.g., granuloma area, fibrosis area in Figs. 2C-D, 3C-D, 8D-E), the number of fields quantified, biological replicates (n), and statistical methods should be clearly stated in the figure legends.

**Response: Thank you for pointing out this critical issue. According to previous reports (DOI: 10.1038/s41586-021-03313-9), we have revised all figure legends—both in the main text and supplementary materials—to clearly specify the number of experimental repeats, biological replicates, and technical replicates. Additionally, we have incorporated this information into the Materials and Methods section and figure legends of the revised manuscript (Page 34, lines 659-662; Page 35, lines 663-666; Page 45, lines 952-954; Page 46, lines 970-972; Page 47, lines 989-991; Page 48, lines 1006-1007; Page 48, lines 1019-1021; Page 50, lines 1046-1048, 1062-1064).**

6. Clinical Correlation Analysis: The use of human transcriptomic data (GSE61376) to show TTP upregulation is a strength. To further enhance the clinical relevance, the authors could explore whether TTP expression levels correlate with established markers of fibrosis severity (e.g., collagen gene expression, AST/ALT levels if available) within this dataset.

**Response: Thank you for this insightful comment. Per your suggestion, the correlation between TTP expression levels and collagen gene expression (*COL1A1*, *COL4A1*, and *TGFB1*) in human transcriptomic data (GSE61376) with schistosomiasis-induced hepatic fibrosis was evaluated by Spearman**

**correlation analysis (new Fig. S1A-C; Page 9, line 139-142; Page 51, lines 1074-1077).**

7. Schematic Diagram: A graphical abstract or a summary schematic model illustrating the proposed TTP-SMAD2/3-WTAP-m6A-TGF-β1/MYB-HSC activation axis would greatly aid readers in visualizing and understanding the complex mechanistic pathway proposed by this study.
**Response: Thank you for this insightful suggestion. As requested, we have added the schematic diagram summarizing the proposed molecular pathway as Figure 9 in the revised manuscript (Page 51, lines 1067-1072).**

Minor Issues:
Language and Grammar: The manuscript requires thorough proofreading to address numerous grammatical errors and improve clarity. While the scientific content is strong, the presentation occasionally hinders comprehension. I strongly recommend revision by a native English speaker or a professional editing service.
Specific examples include:
**Response: Thank you for your valuable feedback. We have had the manuscript thoroughly proofread by a professional English editing service (specialized in biomedical research) to correct grammatical errors, improve clarity, and ensure consistent academic language.**

Line 111-112: The sentence structure is incorrect and the word order is confusing.
Original: "TTP had been identified to negatively regulate TGF-β1 and MYB by activating WTAP transcription, thereby inhibiting HSCs activation S. japonicum-induced in liver fibrosis."
Suggested Revision: "TTP was identified to negatively regulate TGF-β1 and MYB by activating WTAP transcription, thereby inhibiting HSCs activation in S. japonicum-induced liver fibrosis."
**Response: Thank you for your feedback. Using a hepatocyte and HSC co-culture system, our data demonstrate that TTP suppresses HSC activation by negatively regulating TGF-β1 expression. As suggested by you and other reviewers, we have revised the sentence from "TTP had been identified to negatively regulate TGF-β1 and MYB by activating WTAP transcription, thereby inhibiting HSCs activation *S. japonicum*-induced in liver fibrosis" to "TTP was identified to negatively regulate TGF-β1 by activating WTAP transcription, thereby inhibiting HSC activation in *S. japonicum*-induced liver fibrosis."(Page 8, lines 120-122).**

Line 137-139: The word "predominated" is used incorrectly; it should be the adverb "predominantly".
Original: "...indicate that TTP is substantially upregulated predominated in hepatocytes during liver fibrosis."
Suggested Revision: "...indicate that TTP is substantially upregulated, predominantly in hepatocytes, during liver fibrosis."

**Response: Thank you for your feedback. We have implemented the correction according to your advice (Page10, lines 160-161).**

Line 572: There is a likely spelling error in "stereotype".
Original: "Recombinant AAV stereotype vector was a gift..."
Suggested Revision: "Recombinant AAV vectors of serotype 8 (AAV8) were kindly provided by Prof. Y.C. Xia (Wuhan University)."
**Response: Thank you for your feedback. We have implemented the correction according to your advice (Page 35, line 676-677).**
