# Supplementary material for: Tristetraprolin attenuates schistosomiasis-induced liver fibrosis through m⁶A-mediated regulation of TGF-β1 mRNA stability
Source: PLoS Pathog. 2026 May 13;22(5):e1014007. doi: 10.1371/journal.ppat.1014007 (PMC13189414; doi:10.1371/journal.ppat.1014007)
Supplement: Attachment — Submitted filename: Response to Reviewers.pdf [file ppat.1014007.s014.pdf]

Reviewers' Comments:

Reviewer's Responses to Questions

Part I - Summary

Please use this section to discuss strengths/weaknesses of study, novelty/significance, general execution and scholarship.

Reviewer #1: The authors have adequately addressed all concerns raised in my review.

Response: Thank you for your thorough review and valuable feedback. We greatly appreciate the time and expertise you dedicated to evaluating our work—your insights have been instrumental in refining the manuscript.

Reviewer #2: The title of the manuscript has a problem and needs to get fixed. More detail in Part II

Response: We sincerely appreciate your pointing out these issues, and apologize for the redundant "stability" and grammatical errors in the original title. Following Reviewer #3's suggestions, we have revised the title to: Tristetraprolin attenuates schistosomiasis-induced liver fibrosis through m^{6}A-mediated regulation of TGF-β1 mRNA stability (page 1, lines 1-3). The title has been updated consistently throughout the manuscript, and a full check has been conducted to prevent similar errors.

Reviewer #3: This manuscript addresses an important and understudied aspect of host–parasite interaction by implicating tristetraprolin (TTP) and m6A RNA methylation in the regulation of Schistosoma japonicum induced liver fibrosis. The topic is relevant to the journal, and the proposed TTP–WTAP–m6A–TGF-β1 axis is conceptually interesting.

However, several mechanistic and conceptual issues must be addressed before this work can be considered for publication in PLOS Pathogens. In its current form, the manuscript overstates causality, lacks sufficient clarity on epitranscriptomic mechanisms, and does not yet fully demonstrate how parasite-driven pathology specifically engages the proposed pathway.

Major Strengths:

Strong Mechanistic Depth: The multi-layered approach, integrating human data, gain/loss-of-function in vivo models, primary cells, and detailed molecular assays, provides compelling support for the proposed pathway.

Technical Rigor: The use of complementary methods to probe m6A (MeRIP-seq, qPCR, dot blot, pharmacological inhibition) strengthens the conclusions.

Clear Disease Relevance: The study convincingly links the molecular findings to HSC

activation and fibrosis progression in a relevant infection model.
Well-Integrated Discussion: The discussion effectively contextualizes the findings within the broader field, including a balanced analysis of TTP's context-dependent roles.

Areas for Improvement (Clarity and Precision):
The primary issues relate to the precision of language and the completeness of mechanistic narrative, not to the validity of the core findings. Specifically, the manuscript would benefit from: 1) clearer phrasing regarding causal relationships, 2) a more detailed conceptual model for m6A-mediated regulation, and 3) tightened narrative focus. These are matters of presentation and interpretation that can be resolved through textual revisions.

Novelty and Significance:
The study makes a valuable contribution by linking TTP's anti-fibrotic function to the epitranscriptomic machinery (WTAP/m6A) in the context of a parasitic disease. This novel integration of transcriptional co-regulation and post-transcriptional mRNA modification in controlling TGF-β1 output offers fresh insights into the pathogenesis of schistosomiasis liver fibrosis and identifies potential nodes for therapeutic intervention.

Response: Thank you for your insightful and encouraging assessment of our manuscript. We deeply appreciate your recognition of the significance of our work. We agree entirely that enhancing the precision of our language and expanding the mechanistic narrative will significantly strengthen the manuscript. We have carefully considered and addressed all of your concerns, with detailed point-by-point responses below.

Part II – Major Issues: Key Experiments Required for Acceptance
Please use this section to detail the key new experiments or modifications of existing experiments that should be absolutely required to validate study conclusions.

Generally, there should be no more than 3 such required experiments or major modifications for a "Major Revision" recommendation. If more than 3 experiments are necessary to validate the study conclusions, then you are encouraged to recommend "Reject".

Reviewer #1: (No Response)

Reviewer #2: The title has a problem and needs to get fixed: It is currently "TTP alleviates schistosomiasis-induced liver fibrosis via m6A-mediated of regulating TGF-β1 stability mRNA stability" – "stability" is repeated twice and doesn't make sense in general.
In the rebuttal, the authors mention that they changed the title to this title: "TTP alleviates schistosome-induced liver fibrosis via m^{6}A-mediated regulation of TGF-β1 mRNA stability", but this is different from the title that they used in the manuscript.

The authors have addressed all the previous comments and have added a substantial amount of new data to the manuscript.

Response: We sincerely appreciate your pointing out these issues, and apologize for the redundant "stability" and grammatical errors in the original title. Following Reviewer #3's suggestions, we have revised the title to: Tristetraprolin attenuates schistosomiasis-induced liver fibrosis through m^6A-mediated regulation of TGF-β1 mRNA stability (page 1, lines 1-3). The title has been updated consistently throughout the manuscript, and a full check has been conducted to prevent similar errors.

Reviewer #3: The following points require clarification and more precise presentation to fully validate the study's conclusions. These can be addressed through textual modifications and enhanced discussion.

Title and Causality Language: The current title is grammatically problematic. More importantly, throughout the text, some phrasing could be misinterpreted as overstating direct causality (e.g., "TTP protects by increasing m6A"). The data robustly show that m6A is required for TTP's effect. This important distinction should be reflected in a revised title and precise language.

Suggested Action: Adopt a clearer title (e.g., "Tristetraprolin attenuates schistosomiasis-induced liver fibrosis through m6A-mediated regulation of TGF-β1 mRNA stability"). Systematically review the manuscript to ensure causal language accurately reflects the nature of the data, preferring phrases like "m6A-dependent protection" or "associated with."

Response: We sincerely appreciate your comments on the manuscript title and the use of causal language. We have adopted the suggested revised title: Tristetraprolin attenuates schistosomiasis-induced liver fibrosis through m^6A-mediated regulation of TGF-β1 mRNA stability (page 1, lines 1-3). We have also systematically reviewed the entire manuscript, replacing overstated causal expressions with precise phrasing (e.g., "we found that TTP protects against *S. japonicum*-induced liver fibrosis, an effect associated with increased m^6A RNA methylation *in vivo*") to accurately reflect the relationship between TTP and m^6A RNA methylation (page 3, lines 51-53; page 21, lines 406-407).

Mechanism of WTAP Regulation: The statement that "TTP activates the WTAP gene" is a strong claim. While the ChIP-PCR data are supportive, they do not fully distinguish between direct transcriptional activation and indirect effects.

Suggested Action: Modify the language to more precisely describe the observation (e.g., "TTP promotes WTAP expression" or "is associated with increased WTAP transcription"). The Discussion should acknowledge that the exact mechanism (direct vs. indirect) warrants further investigation, which would strengthen the manuscript's scholarly tone.

Response: We sincerely appreciate your insightful comment on the regulatory mechanism of WTAP. We fully agree that the claim "TTP activates the WTAP

gene" is overly strong, as it overstates the scope of our current experimental evidence. Per your suggestion, we have revised the relevant language to more precise phrasing (e.g., "TTP promotes WTAP expression through its interaction with SMAD2/3") throughout the manuscript. Additionally, we have added a specific statement in the Discussion section acknowledging that the current data cannot definitively distinguish between direct and indirect transcriptional regulatory effects of TTP on WTAP, and the precise underlying regulatory mechanism warrants further investigation (page 3, lines 49-51; page 5, lines 74-76; page 8, lines 128-132; page 18, lines 334 and 337; page 19, lines 359-360; page 20, lines 370-371; page 21, lines 393-395; page 23, lines 431-432; page 24, lines 450-452 and 461-470; page 51, line 1086).

Conceptual Model for m6A Function: The manuscript establishes the necessity of m6A but does not elaborate on the logical downstream mechanism. The fate of m6A-modified mRNA depends on "reader" proteins.
Suggested Action: Expand the Discussion to propose a plausible model. Given the observed mRNA destabilization, the authors should discuss the potential involvement of decay-promoting readers (e.g., YTHDF2) and hypothesize how the TTP/SMAD2/3 complex might interface with this step. This conceptual framing will complete the mechanistic story and demonstrate a deeper level of analysis.
Response: We sincerely appreciate this insightful and constructive comment. We fully agree with the well-recognized concept that the fate of m^6A-modified mRNAs is governed by "reader" proteins. However, our luciferase reporter assays showed that YTHDF2 overexpression had no effect on the luciferase activity of the TGF-β1 5′UTR or MYB CDS, suggesting YTHDF2 is unlikely to act as a key "reader" protein for TGF-β1 and MYB. Our data further point to an alternative m^6A recognition mechanism that regulates the mRNA stability of these two genes, with their specific cognate "reader" proteins yet to be identified in future studies. Additionally, immunohistochemical staining revealed elevated YTHDF2 protein levels in the liver tissues of *S. japonicum*-induced liver fibrosis mice. Notably, SMAD2/3 overexpression did not alter YTHDF2 promoter luciferase activity, implying the TTP-SMAD2/3 complex-mediated gene regulatory mechanism characterized in our study does not apply to YTHDF2. Collectively, the molecular mechanisms underlying YTHDF2 upregulation in *S. japonicum*-induced hepatic fibrosis warrant further investigation. We have made corresponding revisions to the Discussion section to enhance the depth of analysis and critical interpretation of our findings (page 24, lines 464-470. page 25, lines 479-483).

Part III – Minor Issues: Editorial and Data Presentation Modifications
Please use this section for editorial suggestions as well as relatively minor modifications of existing data that would enhance clarity.

Reviewer #1: (No Response)

Reviewer #2: The quality of all figures in the main is still very low. All the main figures are blurry/fuzzy. The supplemental figures are sharp and high quality. This is a minor comment.

Response: We sincerely appreciate your careful review and valuable comment. We apologize sincerely for the low-resolution main figures, which likely resulted from document compression during the submission process. We have additionally verified the image size and format requirements specified by the journal to ensure the clarity of the results section in the published version of the manuscript.

Reviewer #3: Addressing these points will enhance the manuscript's clarity, reproducibility, and overall polish.

Abstract & Introduction:

Soften broad therapeutic claims (e.g., "suggests a potential strategy" vs. "provides a rationale for targeting").

Response: Thank you for this valuable feedback. We agree that several of our therapeutic claims were overly broad, and we have accordingly revised the manuscript to refine and soften these statements throughout—particularly within the Abstract and Introduction sections (page 3, lines 53-55; page 4, lines 56-57; page 8, lines 132-133).

Consider de-emphasizing the mention of MYB in the introduction if it is not central to the main narrative, to maintain focus on the TGF-β1 axis.

Response: Thank you for this valuable suggestion. Following your advice, we have further highlighted the TGF-β1 axis in schistosomiasis-induced liver fibrosis in the Introduction (page 6, lines 93-96). Given MYB's involvement in our subsequent experiments and feedback from other reviewers, we have retained one brief statement on MYB's role in hepatic fibrosis here. We hope you will understand.

Clarify in which specific model prior TTP/m6A findings were made to highlight the novelty of the current work.

Response: Thank you for your valuable suggestion. Following your advice, we have revised the Abstract by explicitly emphasizing this study's valuable contribution in linking TTP's anti-fibrotic function to the WTAP/m^{6}A epitranscriptomic machinery in the setting of *S. japonicum*-induced liver fibrosis (page 3, lines 53-55).

Proofread for minor grammatical issues and tighten the final introductory paragraph.

Response: Thank you for your valuable suggestion. We have proofread the entire manuscript for minor grammatical errors and enhanced clarity, and specifically tightened the final introductory paragraph for greater conciseness and impact.

Materials and Methods:

Specify the administration timeline for the inhibitor 3-DAA relative to infection.
Response: Thank you. We have made the revision as per your comment (page 28, lines 530-533).

Briefly note the assessed purity of isolated primary hepatocytes and HSCs.
Response: Thank you for your suggestion.We have supplemented the description on the purity assessment of isolated primary hepatocytes and HSCs in the Materials and Methods section (Page 29, Lines 556–5561). Hepatocytes were verified by their typical polygonal morphology under phase-contrast microscopy (DOI: 10.1186/s13619-025-00258-6). HSCs were identified by their characteristic vitamin A autofluorescence under ultraviolet excitation, a standard method for confirming quiescent HSCs (DOI: 10.1016/j.jhep.2016.01.010). Macrophages were characterized by immunofluorescence staining using an F4/80-specific antibody (DOI: 10.1016/j.imlet.2013.12.002).

Explicitly state the number of biological replicates for MeRIP-seq.
Response: Thank you for your insightful suggestion. For the MeRIP-seq assays, a single biological replicate was employed for each group, consistent with methodologies reported in pertinent prior studies (DOI: 10.1038/s41388-022-02214-z; DOI: 10.1038/s41467-025-60920-0). We integrated MeRIP-seq and RNA-seq data in our analyses, and notably, we observed reduced m6A peaks in the TGFB1 and MYB transcripts of TTP-knockdown cells. Furthermore, we subsequently validated through MeRIP-PCR and luciferase assays that TTP negatively regulates TGF-β1 and MYB expression by enhancing their m6A modification. These relevant details are presented in Figure 4F and Figure S6A.

Add a short statement validating the specificity of key antibodies (custom anti-SMAD2/3, commercial m6A).
Response: We sincerely apologize for any ambiguity arising from our earlier description in the manuscript. Both antibodies are commercially available, and the relevant information regarding their specificity validation has been added to the revised manuscript (page 32, lines 626-627; page 36, lines 691-693).

Results:
Adjust language regarding TTP expression to avoid implying exclusivity to hepatocytes (e.g., "predominantly expressed in...").
Response: Thank you for your valuable suggestion. We have revised the language describing TTP expression throughout the manuscript as advised (page 9, lines 151-152; page 10, lines 168-170; page 25, lines 486-488).

Frame MYB clearly as a secondary target rather than a primary driver equivalent to TGF-β1.

Response: Thank you for your valuable suggestion. We have placed prominent emphasis on TGF-β1 as the primary driver in both the Introduction section and Mechanism Figure 9 of the revised manuscript (page 6, lines 93-96).

When describing the SMAD2/3 interaction, use phrasing like "TTP acts as a transcriptional co-regulator" to avoid misclassification as a canonical transcription factor.
Response: Thank you for your valuable suggestion. In accordance with your advice, we have revised all instances of "co-factor" to "co-regulator" throughout the manuscript (page 5, line 68; page 7, line 121; page 23, line 448).

Discussion:
Consolidate repetitive points about TTP expression patterns into a more concise summary.
Response: Thank you. We have condensed the content on TTP expression patterns in the Discussion section (page 23, lines 435-440).

Replace phrasing like "excessive TTP activates" with more measured terms such as "elevated TTP expression promotes."
Response: Thank you. We have made the revision as per your comment (page 23, lines 431-432).

Add a sentence clarifying the cell-type-specific logic: e.g., "Although TTP is predominantly hepatocytic, its effect on HSC activation is mediated via paracrine TGF-β1 signaling."
Response: Thank you. We have added the indicated sentence to the Discussion section (page 25, lines 486-488).

Ensure the tone in the therapeutic implications section is suitably cautious.
Response: Thank you. We have revised the content on therapeutic implications accordingly to ensure an appropriately cautious tone across the Abstract, Introduction and Discussion sections (page 3, lines 53-55; page 4, lines 56-57; page 8, lines 130-133; page 26, lines 509-512; page 27, lines 519-522).
